# Assessment of 25(OH)D Levels, Their Association with Disease Activity and Nutritional Status in Inflammatory Bowel Disease: A Cross-Sectional Comparative Study

**DOI:** 10.3390/jcm15187044

**Published:** 2026-09-11

**Authors:** Małgorzata Godala, Ewelina Gaszyńska, Izabela Materek-Kuśmierkiewicz, Ewa Małecka-Wojciesko

**Affiliations:** 1 Department of Nutrition and Epidemiology, Medical University of Lodz, 90-752 Lodz, Poland; ewelina.gaszynska@umed.lodz.pl; 2 Department of Internal Medicine and Nephrodiabetology, Medical University of Lodz, 90-419 Lodz, Poland; izabela.materek-kusmierkiewicz@umed.lodz.pl; 3 Department of Digestive Tract Diseases, Medical University of Lodz, 90-153 Lodz, Poland; ewa.malecka-panas@umed.lodz.pl

**Keywords:** inflammatory bowel disease, ulcerative colitis, Crohn’s disease, 25(OH)D, diet, nutritional status, disease activity, nutrition

## Abstract

**Background/Objectives:** Vitamin D deficiency may increase the risk of IBD and be associated with disease activity. The aim of the study was to assess vitamin D levels, their association with disease activity in patients with IBD. **Methods:** A total of 231 subjects took part in the cross-sectional comparative study, including 129 patients with IBD and 102 healthy individuals Disease Activity Index and the Montreal classification were used to assess disease activity in patients with CD. For patients with UC, the Partial Mayo Score and the Montreal classification were applied. To determine total 25(OH)D chemiluminescent immunoassay (CLIA) technology was used. **Results**: The concentration of 25(OH)D was significantly lower in the group of patients with IBD compared to the control group (25.5 ng/mL vs. 28.8 ng/mL, *p* = 0.0027). Differences in 25(OH)D concentrations depended on IBD activity, with significantly higher vitamin D concentrations found in patients in remission compared to those with active IBD (28.9 ± 7.2 ng/mL vs. 22.3 ± 6.1 ng/mL, *p* = 0.0022). This association was confirmed for both UC and CD patients. A multivariate adaptive regression model using spline curves revealed a relationship between serum 25(OH)D concentrations in patients with IBD and total dietary vitamin D intake, including vitamin D supplementation, consumption of one serving of fish per week, and disease remission. **Conclusions**: Serum 25(OH)D levels in IBD patients may serve as an additional, useful, and non-invasive marker of disease activity.

## 1. Introduction

Ulcerative colitis and Crohn’s disease are classified as inflammatory bowel diseases (IBD), which are characterized by a multifactorial etiology with periods of remission and exacerbations [1]. The pathogenesis of IBD is still not fully understood, but among the main factors that interact and play an important role in the development of chronic intestinal inflammation are genetic predisposition, environmental factors, an overactive immune response, and changes in the composition of the gut microbiota [1,2]. Vitamin D treatment associates with decreased disease activity and inflammatory markers and increased IgA-bound and decreased IgG-bound gut microbiota. Vitamin D alters the profiles of IgA-bound (increased *Lachnospiraceae*, *Blautia*) and IgG-bound (decreased *Proteobacteria*, *Enterococcaceae*) gut bacteria. Vitamin D increases B cell activating factor (BAFF) signaling between plasmacytoid dendritic cells and B cells, alters BCR and TCR clonotypes that associate with Ig-bound gut microbiota, and increases α4β7+ B and T regulatory cells. Results of studies demonstrated that vitamin D promotes immune tolerance to gut microbiota in patients with IBD [3,4,5,6].

Results of studies conducted to date have shown that vitamin D deficiency is a common occurrence among IBD patients [3,4,5,6,7,8,9]. The studies have revealed that mean serum 25(OH)D concentrations in patients with IBD were significantly lower compared to healthy individuals, and more than half of the study subjects demonstrated vitamin D deficiency. Causes of the low levels of 25(OH)D in the blood of IBD patients are multifactorial, including lack of exposure to the sun due to immunosuppressive therapy, dietary restrictions, and impaired nutrient absorption [1,2,3,4,5,6].

Intestinal barrier dysfunction plays a key role in IBD, as it leads to the activation of inflammatory signaling pathways, which, in consequence, contributes to intestinal inflammation [7]. Vitamin D is best known as an essential hormone in the regulation of calcium and phosphate metabolism. However, it also plays a key role in regulating the immune response, as evidenced, among other things, by the presence of the VD receptor on immune system cells—T and B lymphocytes, monocytes, and antigen-presenting cells such as macrophages and dendritic cells [2,3,8]. The role of vitamin D in regulating the immune response is reflected in numerous studies on the pathogenesis of autoimmune diseases, including IBD.

Vitamin D plays a key role in maintaining intestinal homeostasis and integrity of the intestinal barrier by stabilizing junctions between epithelial cells, in accelerating the healing process following damage to the intestinal epithelium, and in supporting the balance between the intestinal microbiota and the stimulation of the immune response in the intestines, as well as the development and function of T lymphocytes [9,10,11]. Vitamin D also exhibits antimicrobial activity by increasing the expression of cathelicidin and Human Beta Defensin 2 (HBD2) in phagocytes and epithelial cells [2,12,13]. The barrier function of vitamin D is also linked to its effect on the gut microbiota, and serum levels of vitamin D in humans are correlated with variations in the composition of the gut microbiota and the inflammatory immune response [1,2,14]. Serum of IBD patients who demonstrate a deficiency of one form of vitamin D—25(OH)D3 reveals increased levels of Proteobacteria and decreases levels of Actinobacteria. This observation is in contrast to IBD patients who exhibit optimal levels of the above form of vitamin D. Serum 25(OH)D3 levels are negatively correlated with the amount of certain harmful bacteria, such as Proteobacteria, but positively correlated with the amount of certain probiotic bacteria, i.e., *Lachnospiraceae*, *Bifidobacteriaceae*, and *Anaerostipes* [15,16]. These mechanisms of vitamin D activity are important both in preventing inflammatory bowel disease and in alleviating symptoms of this disease.

According to numerous studies, vitamin D deficiency is common among patients with IBD; it may also increase the risk of IBD and be associated with disease activity and patients’ quality of life [17,18,19,20,21,22]. There are more and more reports regarding potential beneficial effects of vitamin D on the course and incidence of IBD. Hence, vitamin D supplementation—when indicated—is recommended by the European Society for Clinical Nutrition and Metabolism (ESPEN) [23]. However, the optimal level of vitamin D, the method of supplementation, and the relationship between vitamin D deficiency and clinical parameters significant for IBD still require further discussion. Since the results of most studies analyzing calcium-phosphate metabolism in IBD are inconclusive, recent discussions have focused on the potential role of vitamin D as a risk factor for the onset and progression of intestinal inflammation [19,20,21,22,23].

The aim of the study was to assess vitamin D levels, their association with disease activity, and nutritional status in patients, as well as to determine the risk of serum 25(OH)D deficiency in patients with IBD.

## 2. Material and Methods

### 2.1. Study Subjects

A total of 231 subjects took part in the cross-sectional comparative study, including 129 patients with both long-term and recently diagnosed IBD and 102 healthy individuals (the control group). The study group consisted of 75 patients with CD and 54 patients with UC, including 70 women and 59 men aged 18–54 years (mean age 42.7 ± 10.6 years), treated in the Department of Digestive Tract Diseases of the Medical University of Lodz between July 2022 and December 2023. To estimate group size, the G*Power program was used, assuming a medium effect size, an alpha level of 0.05, and a test power of 0.80 (ver. 3.1.9.7; Germany, 2021). The inclusion criteria for patients with IBD were age (≥18 years), and endoscopic and histological diagnosis of CD or UC. The exclusion criteria for all study participants were the presence of neoplastic diseases, kidney disease, metabolic disorders, endocrine disorders, and cancers, as well as prostheses/implants and pacemakers, occurrence of epilepsy, and abnormal conditions of the limbs or trunk (e.g., amputations, scoliosis, skin lesions). The Crohn’s Disease Activity Index (CDAI) and the Montreal classification were used to assess disease activity in patients with CD [24]. For patients with UC, the Partial Mayo Score and the Montreal classification were applied [25]. The control group consisted of clinically healthy individuals without IBD, aged 19–56 years (mean age 44.2 ± 9.7 years), of similar age, sex, smoking status, and BMI.

The study was conducted in accordance with the Declaration of Helsinki, and approved by the Bioethics Committee of the Medical University of Lodz (No. RNN/70/22/KE, 14 June 2022). All the subjects gave written consent to participate in the study.

### 2.2. Blood Tests

Fasting-blood samples were collected from the ulnar vein and used to determine total 25(OH)D using chemiluminescent immunoassay (CLIA) technology. “Liaison 25OH Vit D Total” (LIAISON, Biomedica Gruppe, Vienna, Austria) is a competitive assay based on electrochemiluminescence, employing a specific antibody to identify 25(OH)D2 and 25(OH)D3. A serum 25(OH)D concentration of at least 30 ng/mL was considered normal, while a level below 30 ng/mL was considered insufficient [26]. The 25(OH)D concentrations were assessed according to the season, dividing the year into the winter period (from November to April) and the summer period (from May to October). Total calcium concentrations were determined in compliance with the Olympus Calcium Procedure (reaction of Ca^2+^ ions with the Arsenazo III complex, including albumin); whereas, quantification of inorganic phosphorus was performed with ultraviolet photometry. Calcium/PTH axes were excluded due to laboratory assay variances.

### 2.3. Exposure to UVB Radiation

A self-designed questionnaire was used to assess the subjects’ exposure to UVB radiation. The questionnaire assessed the duration and intensity of sun exposure throughout all seasons, the number of hours spent outdoors per week (8:00 a.m.–4:00 p.m.), and subjects’ preferences regarding spending time exclusively in the sun, both in the sun and in the shade, or only in the shade. The questionnaire also assessed the frequency of using a tanning bed (in the past 3 months), the frequency of visiting countries experiencing the strongest UV radiation levels (in the past 6 months), and the use of UVB filters (never, rarely, frequently, always).

### 2.4. Anthropometry and Physical Activity

All subjects had their height and body weight measured (to the nearest 0.5 cm and 0.1 kg, respectively) with the use of a stadiometer and calibrated medical weighing scales in order to calculate their BMI (Body Mass Index). Underweight was defined for BMI < 18.5 kg/m^2^, normal weight for BMI between 18.5 and 24.9 kg/m^2^, overweight for BMI between 25.0 and 29.9 kg/m^2^, and obesity for BMI ≥ 30.0 kg/m^2^ [27]. In addition, the body composition of the subjects was assessed using bioelectrical impedance analysis (BIA) with the InBody 270 device. The subjects’ levels of fat, water, bone mass, muscle mass, and lean body mass were also determined.

### 2.5. Intake Assessment

The intake of energy and all nutrients was assessed using a thorough 24 h dietary analysis conducted three times for each subject (on two weekdays and one weekend day before the study) by a dietitian. The “Album of Food Products and Dishes” from the Food and Nutrition Institute in Warsaw was used to determine correct portion sizes. The average energy intake as well as the intake of particular nutrients, was assessed using Dieta v. 6.0 software (license no. 52/PD/2022; Institute of Food and Nutrition, Warsaw, Poland) [28].

In order to assess vitamin D intake in the past month, the author used a questionnaire on the frequency of consumption of selected food products that are sources of vitamin D. The products were divided into 7 groups based on the vitamin D content in a standard serving. The number of servings consumed weekly and monthly was determined. The assessed groups of alimentary product included: fresh and smoked fish, fish products, milk and dairy products, eggs, meat and meat products, grain products, and fats. The amount and type of taken supplements were also considered. Study participants who reported taking vitamin D_3_ supplements indicated a daily intake of 1000–2000 IU (25–50 μg) for at least six months prior to the study.

### 2.6. Statistical Analysis

The statistical analysis was performed using STATISTICA v. 13 software (StatSoft Inc., Tulsa, OK, USA). Results are presented using descriptive statistics, including the mean and standard deviation (SD). For nominal scale variables, the frequency of occurrence was calculated. Normality of the distribution of variables was determined using the Shapiro–Wilk test. Results for independent groups were compared using Student’s t-test for quantitative variables and the chi-square test for qualitative variables. Additionally, an One-way ANOVA test was used when the grouping variable had more than two categories. Correlations were assessed using Spearman’s correlation coefficient (R). A *p* value < 0.05 was considered statistically significant. The relationship between the studied factors (total vitamin D intake, fish intake, and normal weight/overweight/obesity) and 25(OH)D concentration in IBD patients was assessed with the use of a multivariate adaptive regression model.

## 3. Results

### 3.1. Characteristics of the Study Subjects

The study group and the control group did not differ significantly in terms of age, gender, BMI, and smoking. No differences were noted in UVB exposure or the use of vitamin D supplements. In both groups, one in five subjects had spent their vacation in a country with strong UV radiation levels within the past 6 months, and nearly 70% of subjects reported spending more than 7 h per week outdoors in the spring and summer. A small percentage of subjects reported that they always used protective measures against UVB. In the IBD patient group, the average duration of the disease was 7.7 ± 5.2 years; 77.5% received biologic therapy, and 41.1% were in clinical and endoscopic remission (Table 1).

### 3.2. Vitamin D Levels and Demographic Characteristics

The mean concentration of 25(OH)D was significantly lower in the group of patients with IBD compared to the control group (25.5 ng/mL vs. 28.8 ng/mL, *p* = 0.0027). However, no significant differences were observed between the groups with regard to the prevalence of vitamin D insufficiency (Table 1).

Among IBD patients, 25(OH)D levels were significantly higher in the group taking vitamin D supplements compared to those who were not taking supplements (30.3 ng/mL vs. 19.7 ng/mL, *p* = 0.0024). The vitamin D levels were significantly higher in the group of subjects who had visited sunny countries within the past 6 months in comparison to those who had not been to such countries (28.7 ng/mL vs. 23.7 ± 5.1 ng/mL, *p* = 0.0031). Furthermore, seasonal fluctuations in 25(OH)D levels were observed in the patient group. Higher 25(OH)D levels were recorded in the summer rather than in the winter (32.1 ng/mL vs. 22.5 ng/mL, *p* = 0.0052). No significant differences in 25(OH)D concentrations were observed in patients with IBD based on the use of UVB protection and time spent outdoors. Similar patterns were observed in the control group (Table 2).

### 3.3. Vitamin D Levels and Nutritional Status

No significant differences in 25(OH)D levels were observed among patients regarding their waist circumference, unintentional weight loss over the past 6 months, or hydration status. However, vitamin D levels did vary among patients based on BMI and body fat content.

Significantly higher 25(OH)D levels were observed in the group of IBD patients with normal BMI values in comparison to the group of underweight patients (29.9 ng/mL vs. 25.6 ng/mL, *p* = 0.0231). Furthermore, vitamin D concentrations were significantly higher in the group of IBD patients with normal BMI values in comparison to the group of overweight patients (29.9 ng/mL vs. 24.7 ng/mL, *p* = 0.0067) as well as in comparison to the patients with obesity (29.9 ng/mL vs. 23.1 ng/mL, *p* = 0.0046).

Similarly to BMI values, body fat percentage also significantly differentiated IBD patients. Significantly higher 25(OH)D concentrations were observed in IBD patients with normal body fat percentage rather than in those with reduced body fat percentage (30.5 ng/mL vs. 22.9 ng/mL, *p* = 0.0124) or those with excessive body fat (30.5 ng/mL vs. 24.1 ng/mL, *p* = 0.0419) (Table 3).

### 3.4. Vitamin D Levels and Disease Activity

Differences in 25(OH)D concentrations depended on IBD activity, with significantly higher vitamin D concentrations found in patients in remission compared to those with active IBD (28.9 ± 7.2 ng/mL vs. 22.3 ± 6.1 ng/mL, *p* = 0.0022). This association was confirmed for both UC and CD patients. Significantly higher vitamin D concentrations were found in UC patients in remission in comparison to those with moderate and severe exacerbations (Partial Mayo Score of 0 vs. 2 and 3; 30.1 ng/mL vs. 22.6 ng/mL vs. 20.9 ng/mL, respectively, *p* = 0.0051). Furthermore, statistically significant differences in 25(OH)D3 concentrations were observed. They depended on the severity of the disease relapse, assessed by the Montreal classification. Thus, higher 25(OH)D concentrations were observed in patients with asymptomatic and mild disease relapses rather than in patients with moderate and severe disease relapses (28.1 ng/mL vs. 27.2 ng/mL vs. 23.2 ng/mL vs. 21.6 ng/mL, *p* = 0.0021) (Table 4).

No significant differences were observed in 25(OH)D concentrations in patients with CD according to disease activity assessed with the use of the Montreal classification; however, significant differences in vitamin D concentrations were noted according to the CDAI. Specifically, higher 25(OH)D levels were found in patients in remission (CDAI < 150) compared to patients with mild, moderate, and severe symptoms (28.1 ng/mL vs. 24.1 ng/mL vs. 23.3 ng/mL vs. 19.9 ng/mL, respectively, *p* = 0.0042) (Table 5).

In the group of patients with IBD, correlation analysis revealed significant relationships between 25(OH)D levels and levels of serum calcium (R = 0.49, *p* < 0.05), phosphorus (R = 0.33, *p* < 0.05), and parathyroid hormone (R = −0.51, *p* < 0.05). The authors reported a correlation between serum 25(OH)D levels and BMI in IBD patients (R = −0.33, *p* < 0.05). In the control group, 25(OH)D concentration was significantly correlated only with parathyroid hormone levels (R = −0.33, *p* < 0.05) and BMI (R = −0.41, *p* < 0.05).

Correlation analysis showed that in the group of IBD patients, 25(OH)D concentration correlated with total dietary vitamin D intake (R = 0.29, *p* < 0.05) and with total dietary calcium intake (R = 0.31, *p* < 0.05). Of all groups of alimentary products, serum 25(OH)D levels in IBD patients positively correlated with fish intake (R = 0.31, *p* < 0.05).

**Figure 1:**
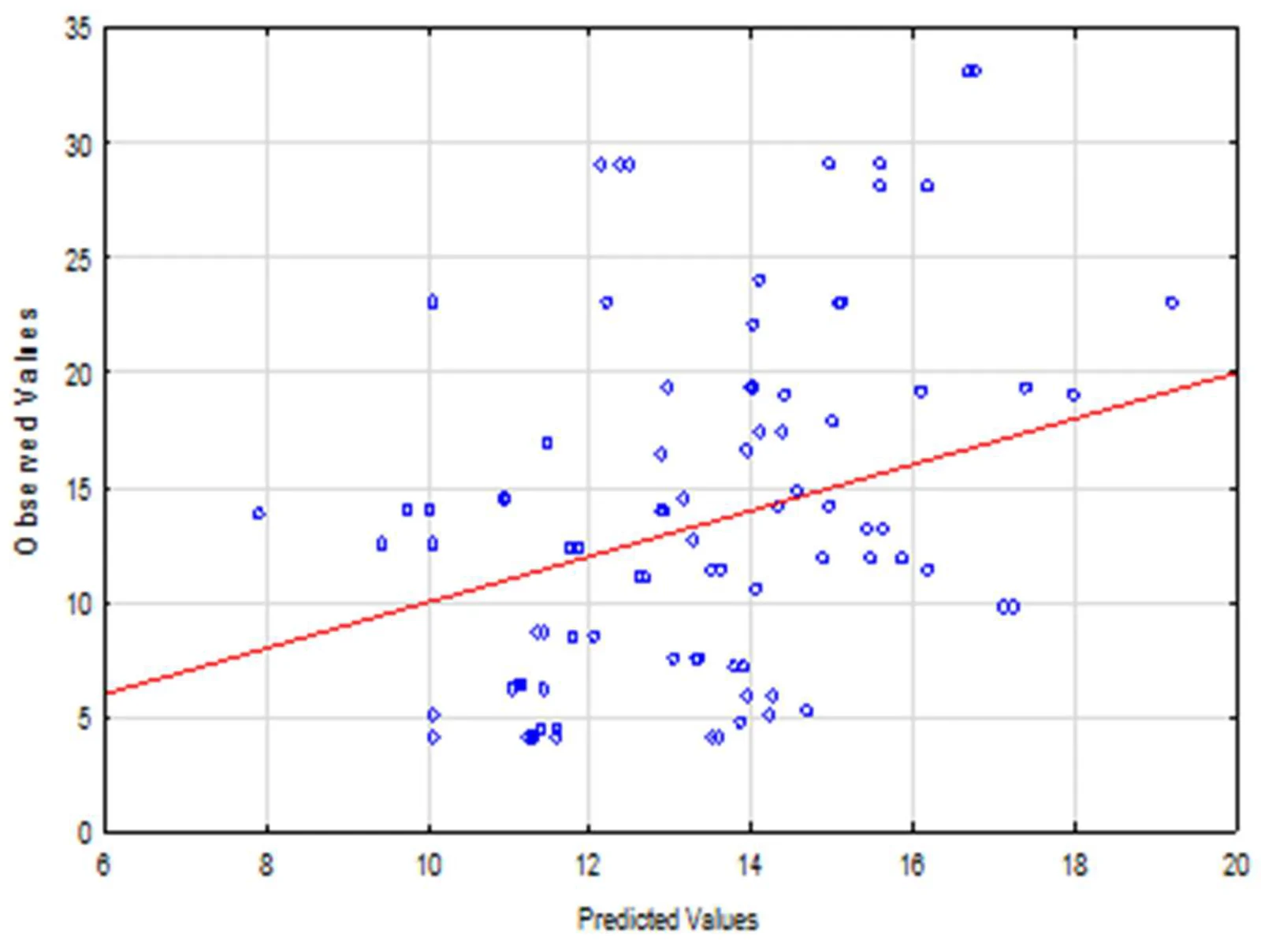
Multivariate adaptive regression model for the serum level of 25(OH)D and its consumption with diet, accounting for intake of vitamin D supplements, consumption of fish, and disease remission.

A multivariate adaptive regression model using spline curves revealed a relationship between serum 25(OH)D concentrations in patients with IBD and total dietary vitamin D intake, including vitamin D supplementation, consumption of 1 serving of fish per week, and disease remission (correlation coefficient R = 0.306, *p* < 0.0001) (Figure 1, Table 6).

## 4. Discussion

In addition to its well-known role in bone mineralization, vitamin D has distinct immunological functions that influence cell proliferation and differentiation, immunomodulation, and the gut microbiome [1,2]. In vitro studies have identified a number of mechanisms through which the biologically active form of vitamin D can reduce inflammation [1]. These observations aroused a lot of interest in the possible pathogenic impact of vitamin D deficiency on the clinical course of IBD, especially as a growing body of evidence suggests that high concentrations of circulating pro-inflammatory biomarkers, such as IL-6, tumor necrosis factor (TNF)-α, and C-reactive protein (CRP), are associated with vitamin D [2,4,18,20]. For this reason, low serum vitamin D levels are suspected to be a risk factor for development of IBD. There are also suggestions that they are also associated with increased disease activity, more frequent relapses, intestinal mucosal inflammation, and reduced quality of life [29]. Seasonal fluctuations in vitamin D levels correlate with seasonal IBD exacerbations, which peak in the spring following months of reduced vitamin D production due to insufficient sunlight [29,30,31].

In our study, we demonstrated vitamin D insufficiency in more than half of IBD patients and significantly lower serum concentrations in patients in comparison to healthy individuals.

Professional literature contains study results that confirm our observations. De Souza et al. found significantly lower serum 25(OH)D concentrations in patients with CD and UC compared to healthy individuals. Vitamin D deficiency was observed in almost half of the studied IBD patients [8]. Ananthakrishnan et al. demonstrated 25(OH)D deficiency in more than half of IBD patients and further confirmed a higher risk of cancer, particularly colorectal cancer, in patients with vitamin D deficiency [32]. Meckel et al. demonstrated insufficient mean serum 25(OH)D concentrations in UC patients, and the recommended vitamin D level was found in only 12.6% of patients [33]. Dumitrescu et al., in a study on vitamin D nutritional status in IBD patients, found significantly lower 25(OH)D concentrations in patients with CD and UC compared to healthy individuals. The authors revealed that only 24% of UC patients and 21% of CD patients demonstrated normal serum 25(OH)D levels [34]. Mechie et al. also confirmed serum 25(OH)D deficiency in more than half of the studied IBD patients [17]. A 2019 meta-analysis of 55 studies evaluating vitamin D status in IBD patients confirmed that 25(OH)D concentrations are significantly lower in patients than in healthy subjects, and that vitamin D deficiency is common in this patient group [6].

In this study, we demonstrated a relationship between vitamin D status and disease activity. We found significantly lower serum 25(OH)D concentrations in patients during disease relapse than during remission, for both CD and UC. Literature reports are not conclusive. Yet, most studies have confirmed our findings. Hausmann et al. assessed serum vitamin D levels in patients with IBD. They noted that vitamin D deficiency is common in this group of patients and inversely correlated with disease activity, in both the CD and UC groups [35]. Similar findings were obtained in a German study, which demonstrated significantly lower vitamin D levels in patients during periods of exacerbated IBD symptoms compared to those in remission. This association was confirmed for both CD and UC patients [17]. Slightly different results were obtained in a study conducted in a group of IBD patients in the Romanian population. The authors observed a relationship between disease activity and serum vitamin D levels exclusively in patients with CD. This relationship was not confirmed for patients with UC [34]. In contrast, a study by Zielińska et al. found no association between serum 25(OH)D levels and the activity of CD and UC. Furthermore, the authors reported higher serum vitamin D levels in patients compared to healthy individuals [36].

Lower serum vitamin D levels in patients with IBD, especially during periods of exacerbations, may result from several factors. First, IBD is associated with chronic inflammation and, consequently, malabsorption syndromes, as confirmed by numerous studies assessing the nutritional status of IBD patients, including vitamin and mineral status [17,37,38]. Furthermore, exacerbations are often accompanied by a lower dietary intake of energy and nutrients, as well as greater losses of these due to diarrhea. Geographic and cultural location itself is also important due to cutaneous synthesis and high consumption of foods rich in vitamin D, such as fish and seafood, which can be observed in certain regions. In this study, we demonstrated an association between serum 25(OH)D levels and dietary vitamin D intake, particularly including supplementation and fish consumption at least once a week. On the other hand, we also demonstrated the impact of cutaneous synthesis on vitamin D status in patients with IBD, which confirms the seasonal pattern of changes in serum 25(OH)D concentrations and the effect of UV exposure during stays in countries with high ultraviolet radiation.

Vitamin D supplementation prevents development of or alleviates the body’s inflammatory response and may help to minimize disease progression [39]. A study by Domislovic et al., conducted on the Croatian IBD population, showed that patients with inflammatory bowel disease and vitamin D deficiency are at an increased risk of hospitalization, surgical procedures, and loss of response to biologic therapy, while high vitamin D levels are considered a protective factor [40]. In their meta-analysis, Guo et al. demonstrated a statistically significant inverse relationship between oral vitamin D supplementation and serum CRP levels [41]. However, determining a safe and recommended dose of vitamin D for patients with IBD poses a challenge and is the subject of many studies [42,43,44]. A gold standard of care has still not been established; however, most literature suggests oral supplementation rather than other routes of administration, as the first route allows to minimize potential side effects of the therapy. In a randomized study, Kojecky et al. demonstrated comparable efficacy of continuous vitamin D supplementation at a dose of 2000 IU/day in the fall and winter compared to a dose adjusted to body weight (28 IU/kg body weight) [42]. It has also been demonstrated that the response to infliximab treatment in patients with IBD and vitamin D deficiency is poorer than in patients with normal serum vitamin D levels, and patients most often discontinue therapy as they cannot see any health improvement [45]. The issue of whether vitamin D supplementation can induce remission also requires further discussion.

In our study, we also demonstrated an association between serum 25(OH)D concentration and patients’ nutritional status, as assessed by BMI and body fat content. The highest vitamin D concentrations were observed in patients with normal BMI and normal body fat content. Interestingly, both overnutrition and undernutrition, as assessed by these indicators, resulted in lower serum 25(OH)D concentrations. In professional literature, there are hardly any studies evaluating vitamin D status in IBD patients in the context of nutritional status. Pappa et al. demonstrated that vitamin D deficiency is very common in pediatric patients with inflammatory bowel disease. This study also showed that predisposing factors for vitamin D deficiency include dark skin tone, the fall-winter season, lack of vitamin D supplementation, early stage of the disease, a more severe disease course, and upper gastrointestinal tract involvement in patients with CD. This study also confirmed a positive correlation between 25(OH)D levels and BMI values [46]. A study by Pallav et al., conducted on 237 patients with IBD, revealed lower 25(OH)D levels in patients with BMI above 30 kg/m^2^, indicating thereby that obesity is a predisposing factor for vitamin D deficiency in this patient group [47]. Tajika et al. reached similar conclusions, identifying obesity as a risk factor for vitamin D deficiency in patients with CD and UC [48].

Results of our study and those carried out by other authors confirm the association between vitamin D status in IBD patients and nutritional status. Lower serum 25(OH)D concentrations in obese patients are probably related to the sequestration of vitamin D in adipose tissue. This process consists of uptake and storage of lipophilic vitamin D in fat cells, which allows for a release of vitamin D into the bloodstream in much smaller amounts. This phenomenon is the main cause of widespread vitamin D deficiency in overweight and obese individuals. In contrast, the lower serum 25(OH)D concentrations we observed in malnourished patients are likely related to insufficient dietary intake of vitamin D and/or increased losses of the vitamin. Regardless of disturbances in nutritional status, maintaining a healthy body weight is, alongside dietary intake and proper cutaneous synthesis, one of the key factors in providing sufficient vitamin D status in patients with IBD.

Considering the fact that pathophysiology of IBD is still unclear, the search for new markers to assess the activity of this disease seems reasonable. Furthermore, new markers may improve treatment outcomes. Serum 25(OH)D is a micronutrient with significant metabolic implications. Its deficiency can lead to severe extraintestinal complications, such as osteoporosis or fractures. It is also worth stressing that vitamin D deficiency is easy to diagnose and can be corrected with appropriately selected supplementation, thereby reducing the incidence of complications and improving remission rates, which is clinically significant. It seems reasonable to recommend that patients have their 25(OH)D levels measured at least once a year, especially in the fall and winter, as well as initiate supplementation. These measures may contribute to clinical remission, thereby leading to better treatment outcomes and reduced healthcare costs.

Literature reports highlighting the role of vitamin D in various aspects of the regulation of the body’s immune response suggest that the vitamin should be considered not only as a vitamin involved in calcium-phosphate homeostasis, but also as an autocrine and paracrine mediator actively involved in the pathomechanism of many diseases, including IBD. Our study indicates that monitoring vitamin D levels in this patient group appears to be an important issue. In clinical practice, patients with IBD should supplement their diet with vitamin D and be aware of the impact of different seasons on its serum levels. In the light of available studies, it can be assumed that vitamin D may be used in IBD treatment as an adjunctive therapy.

Our study has certain limitations. It is a single-center study and, for this reason, may not be representative for all IBD patients. It was conducted on a relatively small group of patients, and the control group consisted exclusively of healthy individuals. Some information provided by patients, like UVB protection, may not be objective. Chemiluminescent Immunoassays are known to be less accurate than Liquid Chromatography/Tandem Mass Spectrometry, which is the established reference standard in clinical chemistry. Since blood samples were collected only once, it was not possible to analyze the impact of seasonal variations, changes in diet, or the impact of supplementation over time for each patient. The relationship between nutritional status, disease activity, and serum 25(OH)D may be influenced by systemic inflammation.

## 5. Conclusions

Serum 25(OH)D levels in IBD patients may serve as an additional, useful, and non-invasive marker of disease activity, and normalization of these levels may reduce the incidence of complications and improve remission rates, which is clinically significant. However, vitamin D deficiency may be a consequence rather than a cause of active inflammation. Therefore incorporating serum 25(OH)D as a potential IBD activity biomarker requires further research.

## Figures and Tables

**Table 1:** Characteristics of the study participants.

	IBD *n* = 129	Controls *n* = 102	*p*
Age [years]	42.7 ± 10.6	44.2 ± 9.7	0.421
CD [*n*,%]	75 (58.1)	-	-
Female [*n*,%]	70 (54.3)	56 (54.9)	0.573
Body mass index BMI [kg/m^2^]	24.7 ± 5.1	26.6 ± 3.5	0.738
Regular vitamin D supplements users [*n*,%]	37 (28.7)	33 (32.4)	0.171
Biology therapy [*n*,%]	100 (77.5)	-	-
Immunosuppression [*n*,%]	50 (38.8)	-	-
Steroids [*n*%]	47 (36.4)	-	-
5-ASA [*n*,%]	39 (30.2)	-	-
Smoking [*n*,%]	15 (11.6)	12 (11.7)	0.845
Disease duration [years]	7.7 ± 5.2	-	-
Surgery history [*n*,%]	26 (20.2)	-	-
Remission [*n*,%]	53 (41.1)	-	-
Disease activity CD			
CDAI [0/1/2/3]	26(34.7)/16(21.3)/27(36)/6 (8)	-	-
Montreal classification			
Age at diagnosis [A1/A2/A3]	14(18.7)/56 (74.6)/5(6.7)	-	-
Disease location [L1/L2/L3]	27(36)/11(14.7)/37(49.3)	-	-
Disease behavior [B1/B2/B3]	32(42.6)/28(37.4)/15(20)	-	-
Disease activity UC			
Partial Mayo Score [0/1/2/3]	27(50.0)/0(0)/17(31.5)/10(18.5)	-	-
Montreal classification			
Disease location [E1/E2/E3]	6(11.1)/25(46.3)/23(42.6)	-	-
Severity of relapse [S0/S1/S2/S3]	17(31.5)/16(29.6)/16(29.6)/5(9.3)	-	-
**UVB exposure**			
Sunny vacation during the previous 6 months [*n*,%]	26 (20.2)	21 (20.6)	0.7649
Outside during daylight > 7 h/week [*n*,%]	88 (68.2)	69 (67.6)	0.6548
Summer [*n*,%]	81 (62.8)	68 (66.7)	0.7592
Winter [*n*,%]	48 (37.2)	34 (33.3)	0.5789
**Sun-protection products**			
Never [*n*,%]	44 (34.1)	32 (31.4)	0.7458
Rarely [*n*,%]	30 (23.2)	26 (25.5)	0.6852
Often [*n*,%]	42 (32.6)	29 (28.4)	0.7647
Always [*n*,%]	13 (10.1)	15 (14.7)	0.6179
**Dietary intake**			
Energy [kcal/day]	1627.7 ± 427.1	2227.4 ± 982.8	<0.0001
Fats [g/day]	56.4 ± 21.3	77.3 ± 61.2	<0.0001
Proteins [g/day]	56.9 ± 18.7	95.6 ± 47.3	<0.0001
Carbohydrates [g/day]	173.0 ± 59.4	257.8 ± 131.9	<0.0001
Vitamin D (without supplements) [μg/day]	3.8 ± 2.2	7.1 ± 4.3	<0.0001
Total vitamin D (including supplements) [μg/day]	37.2 ± 14.1	21.7 ± 14.1	0.0028
Calcium (without supplements) [mg/day]	572.5 ± 258.3	688.5 ± 466.3	<0.0001
Total calcium (including supplements) [mg/day]	743.1 ± 411.2	677.8 ± 396.3	<0.0001
Phosphorus [mg/day]	973.3 ± 316.2	1689.2 ± 755.1	<0.0001
**Groups of products**			
Fresh and smoked fish [portions/week]	1.3 ± 0.6	2.2 ± 1.2	<0.0001
Fresh and smoked fish [μg vitamin D/week]	10.2 ± 3.1	20.8 ± 6.3	<0.0001
Fish products [portions/week]	1.2 ± 0.7	2.8 ± 0.8	<0.0001
Fish products [μg vitamin D/week]	13.9 ± 2.3	25.9 ± 5.9	<0.0001
**Serum concentration**			
25(OH)D total [ng/mL]	25.5 ± 9.8	28.8 ± 7.9	0.0027
25(OH)D insufficiency [*n*,%]	85 (65.9)	64 (62.7)	0.8116
Calcium [mmol/L]	2.5 ± 0.4	2.3 ± 0.3	0.8289
Phosphorus [mmol/L]	1.1 ± 0.6	1.2 ± 0.6	0.8728

**Table 2:** Concentrations of 25(OH)D in study participants.

	25(OH)D [ng/mL]	*p*
**IBD**		
CD	25.4 ± 8.9	0.7756
UC	26.1 ± 10.5	
Active	22.3 ± 6.1	0.0022
Remission	28.9 ± 7.2	
Women	25.7 ± 8.5	0.8678
Man	26.6 ± 7.7	
Supplementation	30.3 ± 7.2	0.0024
Without supplementation	19.7 ± 6.9	
UVB protection	27.1 ± 7.7	0.7784
Without UVB protection	24.9 ± 8.1	
>7 h outside/week	26.6 ± 6.1	0.8816
<7 h outside/week	24.8 ± 7.2	
Sunny vacation	28.7 ± 3.3	0.0031
Without sunny vacation	23.7 ± 5.1	
Summer	32.1 ± 6.7	0.0052
Winter	22.5 ± 8.2	
**Controls**		
Women	27.8 ± 7.9	0.7451
Man	26.9 ± 9.2	
Supplementation	32.1 ± 7.1	0.0115
Without supplementation	25.1 ± 8.2	
UVB protection	28.8 ± 6.9	0.8835
Without UVB protection	29.1 ± 7.8	
>7 h outside/week	27.9 ± 8.1	0.8021
<7 h outside/week	28.7 ± 7.7	
Sunny vacation	30.9 ± 7.2	0.0127
Without sunny vacation	25.6 ± 8.8	
Summer	33.1 ± 7.7	0.0032
Winter	24.1 ± 9.2	

**Table 3:** Concentrations of 25(OH)D according to nutritional status of patients with IBD.

	25(OH)D [ng/mL] Mean ± SD	*p*
**BMI [kg/m^2^]**		
<18.5	25.6 ± 7.3	0.0041 * 0.0447 ***
18.5–24.9	29.9 ± 8.7	
>25	24.7 ± 8.9	
>30	23.1 ± 7.9	
**Waist circumference [cm]**		
Normal	26.9 ± 8.1	0.5497
High	24.6 ± 6.9	
**Unintentional weight reduction in 6 months**		
<10	24.9 ± 7.8	0.4873
>10	27.1 ± 9.2	
**Adipose tissue [%]**		
Low	22.9 ± 8.6	0.0412 ** 0.0051 ***
Normal	30.5 ± 7.2	
High	24.1 ± 8.9	
**Water content [%]**		
Low	26.9 ± 9.1	0.7751
Normal	24.8 ± 9.8	

* Univariate analyses. Post hoc multiple comparisons. <18.5 vs. 18.5–24.9 *p* = 0.0231; <18.5 vs. >25 *p* = 0.4668; <18.5 vs. >30 *p* = 0.4411; 18.5–24.9 vs. >25 *p* = 0.0067; 18.5–24.9 vs. >30 *p* = 0.0046; >25 vs. >30 *p* = 0.7492. ** Univariate analyses. Post hoc multiple comparisons: low vs. normal *p* = 0.0124; low vs. high *p* = 0.8772; normal vs. high *p* = 0.0419. *** Multivariate analyses involved all the above-mentioned factors, stratifying by CD vs. UC and controlling for age and gender.

**Table 4:** Concentrations of 25(OH)D according to UC activity.

	25(OH)D Mean ± SD
**Partial Mayo Score**	
0	30.1 ± 7.7
2	22.6 ± 6.9
3	20.9 ± 7.1
*p*-value	0.0051 *
**Montreal Classification**	
E1	26.1 ± 9.1
E2	24.4 ± 8.1
E3	24.9 ± 5.5
*p*-value	0.2218
S0	28.1 ± 5.7
S1	27.2 ± 5.9
S2	23.2 ± 6.7
S3	21.6 ± 7.3
*p*-value	0.0021 *

* Univariate analyses. Post hoc multiple comparisons. Mayo Score 0 vs. 2 *p* = 0.0342; 0 vs. 3 *p* = 0.0091; 2 vs. 3 *p* = 0.7552; S0 vs. S1 *p* = 0.1749; S0 vs. S2 *p* = 0.0071; S0 vs. S3 *p* = 0.0525; S1 vs. S2 *p* = 0.0076; S1 vs. S3 *p* = 0.0172; S2 vs. S3 *p* = 0.6579.

**Table 5:** Concentrations of 25(OH)D according to CD activity.

	25(OH)D Mean ± SD
**Age at onset (years)**	
A1 < 16	23.3 ± 8.1
A2 17–40	23.9 ± 7.7
A3 > 40	25.7 ± 7.3
*p*-value	0.6528
**Localization**	
L1 Ileum	24.1 ± 6.6
L2 Colon	25.1 ± 8.1
L3 Ileum + colon	23.8 ± 5.7
*p*-value	0.7335
**Course of the disease**	
B1 No stenoses or fistulas	24.7 ± 7.2
B2 Stenoses	23.9 ± 7.5
B3 Fistulas	25.6 ± 5.9
Perianal lesions	25.9 ± 6.7
*p*-value	0.1123
**CDAI**	
<150	28.1 ± 7.1
150–220	24.1 ± 6.6
221–450	23.3 ± 6.8
>450	19.9 ± 7.9
*p*-value	0.0042 *

* A univariate analysis. Post hoc multiple comparisons. CDAI < 150 vs. CDAI 150–220, *p* = 0.0272; CDAI < 150 vs. CDAI 221–450, *p* = 0.0209; CDAI < 150 vs. CDAI > 450, *p* = 0.0023; CDAI 150–220 vs. CDAI 221–450, *p* = 0.7721; CDAI 150–220 vs. CDAI > 450, *p* = 0.2343; CDAI 221–450 vs. CDAI > 450, *p* = 0.4231.

**Table 6:** Multivariate adaptive regression model for the serum level of 25(OH)D and its consumption with diet, accounting for the intake of vitamin D supplements, consumption of fish, and remission.

Vitamin D [ng/mL]	Correlation Coefficient	F-Test	df	Standard Error of Estimate	*p*-Value
	0.306	3.418	4.132	7.037	<0.0001
Vitamin D supplements intake	0.27				<0.0001
Consumption of fish [1 portion/week]	0.26				<0.0001
Disease remission	0.12				<0.0001

df—degrees of freedom.

## Data Availability

The original contributions presented in this study are included in the article. Further inquiries can be directed to the corresponding author.
